# A case report on IDH-mutant astrocytoma, CNS WHO grade 4: multi-omic characterization of untreated clinical progression

**DOI:** 10.3389/fonc.2025.1557245

**Published:** 2025-09-26

**Authors:** Xiaoqin Tang, Wei Wei, Yanyan Huang, Yi Zheng, Yajing Xu, Fei Li

**Affiliations:** Department of Neurosurgery, Southwest Hospital, Chongqing, China

**Keywords:** disease progression, glioma, astrocytoma, grade 4, isocitrate dehydrogenase, tumor-infiltrating lymphocytes, case reports

## Abstract

**Background:**

The natural history of untreated IDH-mutant astrocytoma, CNS WHO grade 2, progressing to astrocytoma grade 4 remains poorly characterized.

**Methods:**

A 67-year-old woman with a histologically confirmed grade 2 astrocytoma developed a spatially adjacent grade 4 lesion after eight years without therapeutic intervention. Tumor tissue and peripheral blood were analyzed using integrated genomic, transcriptomic, and immune profiling.

**Results:**

The central region retained grade 2 histology, while the peripheral region exhibited grade 4 features. Both shared mutations in *IDH1*, *TP53*, and *ATRX*, with highly concordant methylation patterns. The grade 4 lesion uniquely acquired mutations in *CIC*, *BRCA2*, and *RPA4*, and showed a 70% increase in *NAF1* mutant allele frequency. Pathway analysis revealed MSP-RON and NF-κB activation, increased mast cell infiltration, and reduced IL-17 signaling, dendritic cells, and CD4^+^/CD8^+^ T-cell presence. Among the 1,926 peripheral blood T-cell receptor clonotypes, only 2.1% were detected in the tumor regions. Two highly abundant clonotypes were consistently present in peripheral blood, grade 2, and grade 4 samples, indicating clonal persistence across compartments.

**Conclusion:**

This case highlights the clonal progression of an IDH-mutant astrocytoma from grade 2 to grade 4, potentially driven by additional mutations and immune remodeling. These exploratory findings suggest candidate mechanisms of glioma evolution and may inform adoptive T-cell therapy approaches.

## Introduction

Diffuse low-grade gliomas, particularly isocitrate dehydrogenase (IDH)-mutant astrocytomas, frequently progress to higher-grade forms such as astrocytoma, CNS WHO grade 4. Despite advances in molecular profiling and the adoption of the 2021 WHO classification, the biological mechanisms underlying this malignant transformation remain poorly understood, especially in the absence of therapeutic interference (1). Previous studies have implicated multiple pathways in astrocytoma progression, including PI3K/Akt/mTOR signaling (2), RB pathway disruption (3, 4), dysregulated cell cycle control (5), DNA methylation reprogramming (6), and MET-exon-14-skipping (METex14) (7). In addition, gliomas develop a profoundly immunosuppressive tumor microenvironment, in which impaired local immune surveillance may facilitate malignant progression (8–10). However, current understanding is primarily derived from tumors that have undergone surgery, chemotherapy (11), or radiotherapy (12), which confound the natural history of progression. Here, we report a rare case of untreated IDH-mutant astrocytoma that progressed from CNS WHO grade 2 to grade 4 over eight years, without resection or effective adjuvant therapy, providing a unique opportunity to explore intrinsic mechanisms of glioma evolution under unaltered clinical conditions.

## Case presentation

**Figure 1:**
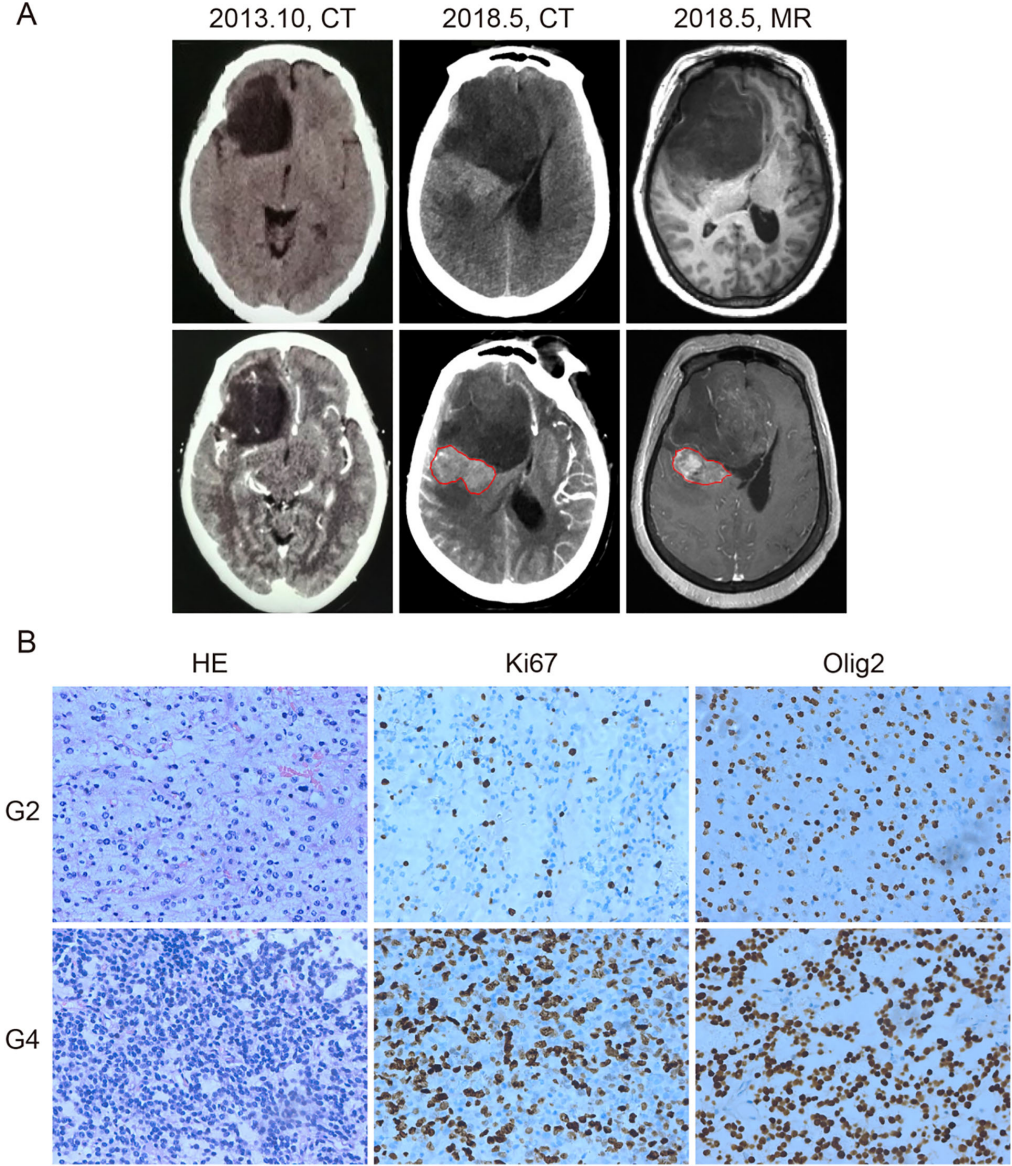
Computed tomography (CT), magnetic resonance imaging (MRI), and histological diagnosis of the patient. **(A)** CT and MRI images of the tumor. In October 2013, CT showed a low-density area in the right frontal lobe without enhancement (left panel). By May 2018, CT (middle panel) and contrast-enhanced MRI (right panel) revealed tumor enlargement and a newly enhancing region (outlined in red), suggesting malignant progression. **(B)** Hematoxylin and eosin (HE), anti-Ki67, and anti-Olig2 staining of the grade 2 (G2) and grade 4 (G4) tumor samples. Grade 4 tissue exhibited higher cellular density and increased Ki67 staining, indicating elevated proliferative activity. Olig2 positivity was retained in both grades. Brown nuclear staining indicates positive cells. Images are representative of observed patterns.

A 67-year-old woman presented in June 2010 with persistent headaches and dizziness. Initial brain computed tomography (CT) identified an intracranial lesion. Follow-up imaging in October 2013 showed a non-enhancing, low-density area in the right frontal lobe with a suborbicular appearance, suggesting a low-grade glioma. The patient remained untreated until May 2018, when she developed worsening headaches, blurred vision, and left-sided weakness. Repeat CT and magnetic resonance imaging (MRI) demonstrated significant tumor enlargement, midline shift due to lateral ventricular compression, and a newly enhancing region posterior to the original lesion (**Figure 1A**), raising suspicion for high-grade transformation.

On June 1, 2018, the patient underwent surgical resection of the entire lesion. Histopathological and molecular analyses revealed two distinct regions: a central component consistent with astrocytoma, IDH-mutant, CNS WHO grade 2, and a peripheral component corresponding to astrocytoma, IDH-mutant, CNS WHO grade 4, according to the 2021 WHO Classification of Tumors of the Central Nervous System (8). The grade 4 region showed increased cellularity, a higher Ki-67 proliferation index, and stronger Olig2 expression compared to the grade 2 region, suggesting a more aggressive biological phenotype (**Figure 1B**).

### Untreated clinical progression from grade 2 to grade 4 astrocytoma

**Figure 2:**
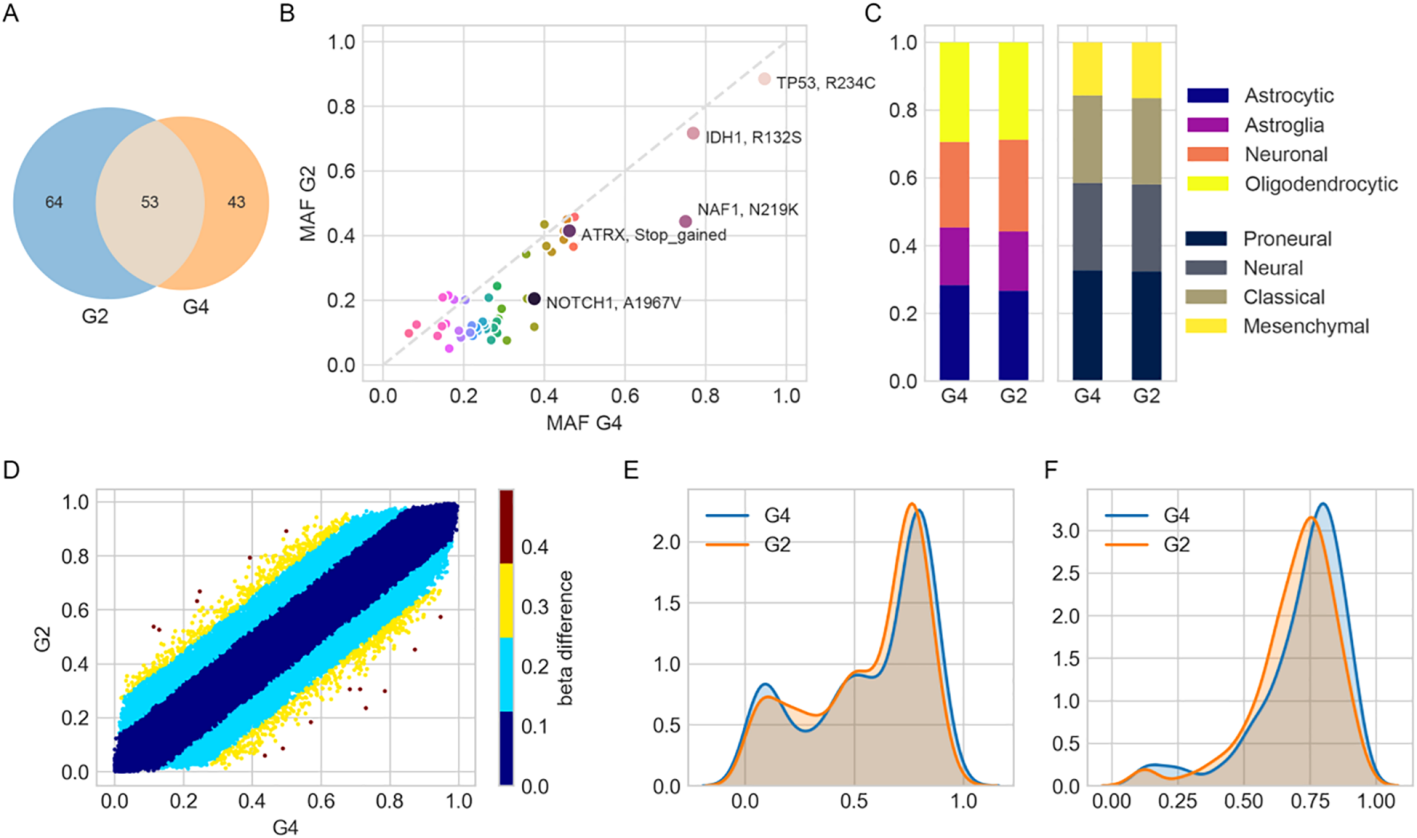
Molecular and transcriptional characterization of grade 2 (G2) and grade 4 (G4) astrocytoma regions. **(A)** Venn diagram showing the number of unique and shared mutations identified in G2 and G4 tumor samples. **(B)** Scatterplot comparing minor allele frequencies (MAFs) of shared mutations between G2 and G4. **(C)** Bar plots showing relative enrichment scores of cell-type and transcriptional subtype signatures derived from ssGSEA using previously published gene sets. **(D)** Scatterplot of DNA methylation β-values for shared probes between G2 and G4. Color intensity reflects the magnitude of β-value difference, indicating changes in methylation. **(E, F)** Kernel density plots of β-values for probes defining **(E)** CpG island methylator phenotype (CIMP, 1,503 probes) and **(F)** CIMP-high subtype (131 probes), illustrating epigenetic landscape differences.

A total of 117 and 96 somatic mutations were identified in the grade 2 and grade 4 astrocytoma regions, respectively, with 53 mutations shared between them (**Figure 2A**). Canonical glioma-associated alterations such as *TERT* promoter mutations (C228T, C250T), *EGFR* VIII fusion, and 1p/19q codeletion were absent. Mutations in *IDH* (R132S), *TP53* (R234C), and *ATRX* (T1582NfsTer19) were observed in both regions, with comparable mutant allele frequencies (MAFs). Among them, *IDH* and *TP53* exhibited MAFs exceeding 70%, supporting their classification as early mutations present in both tumor regions (**Figure 2B**).

Transcriptomic subtype analysis based on the Verhaak classification (9) revealed proneural enrichment in both samples (normalized scores: 32.4% in grade 2, 32.8% in grade 4), along with mixed subtype characteristics reflecting intra-tumoral heterogeneity. Cell-of-origin deconvolution indicated contributions from oligodendrocyte- and astrocyte-like gene signatures (28.7% and 29.4% in grade 2, 26.6% and 28.3% in grade 4, respectively) (**Figure 2C**), which may reflect microenvironmental influences rather than definitive cellular lineage.

In DNA methylation profiling, β-values range from 0 (unmethylated) to 1 (fully methylated). Values around 0.75 indicate extensive promoter hypermethylation across CpG islands. Such a pattern is characteristic of the CpG Island Methylator Phenotype (CIMP), which has been associated with distinct epigenetic tumor subtypes and potentially poorer prognosis (10). DNA methylation profiles were highly concordant between grade 2 and grade 4 (Pearson R² = 0.991). Only 4,035 out of 761,759 CpGs exhibited β-value differences greater than 0.2 (**Figure 2D**). Both samples showed a high probability of *MGMT* promoter methylation (0.99) and were classified as CpG island methylator phenotype (CIMP)^+^, with peak β-values around 0.76 (**Figures 2E, F**).

**Figure 3:**
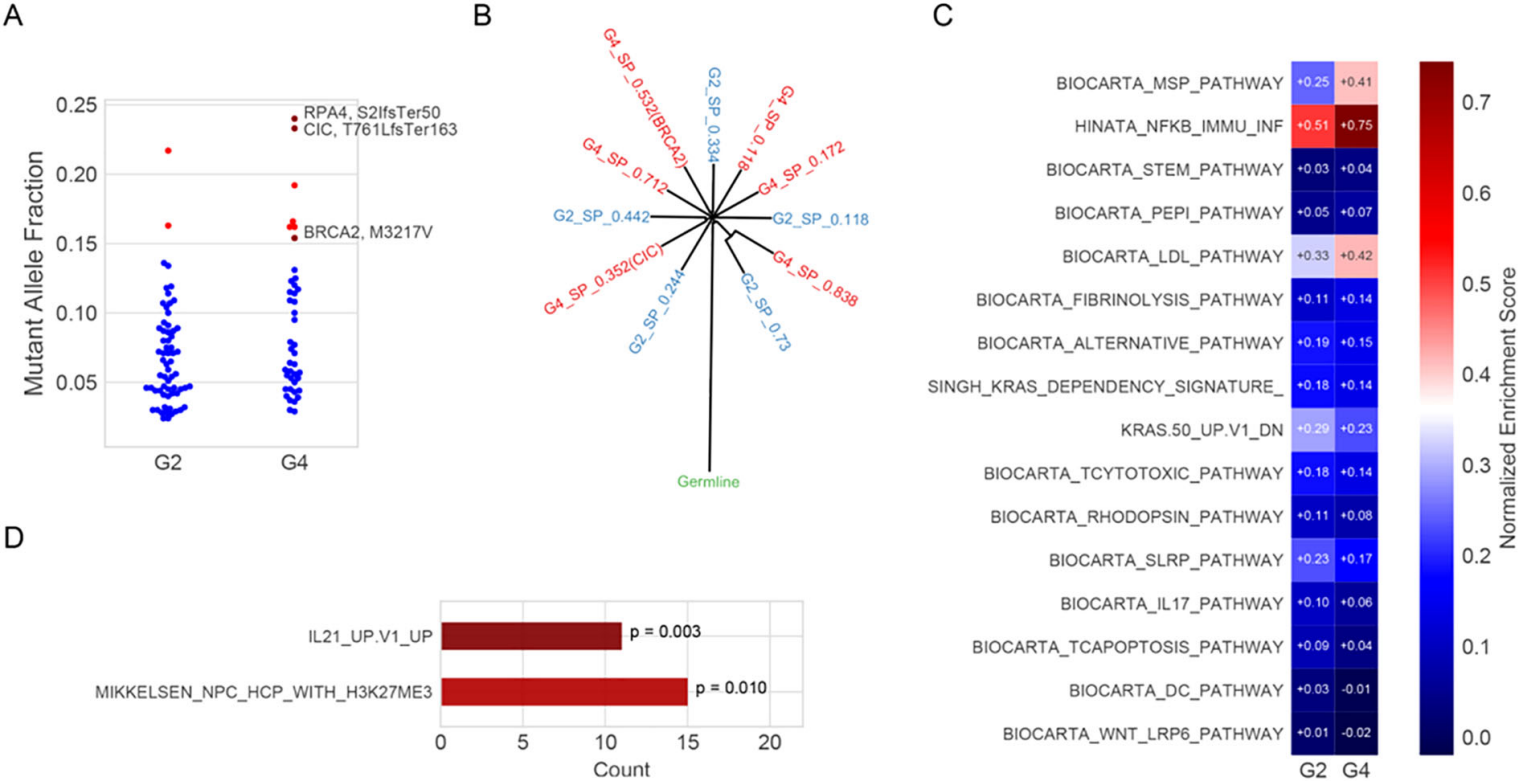
Clone evolution and enrichment pathway analysis. **(A)** Mutant allele fractions (MAFs) of somatic mutations in G2 and G4 regions. Blue dots represent unannotated variants; red triangles indicate mutations potentially associated with malignant progression (e.g., *RPA4* S21fsTer50, *CIC* T761LfsTer163, and *BRCA2* M3217V). *RPA4* (S21fsTer50): serine at position 21 to frameshift, terminating at position 50; *CIC* (T761LfsTer163): threonine at position 761 to leucine frameshift, terminating at position 163; *BRCA2* (M3217V): methionine at position 3217 substituted by valine. **(B)**. Inferred clonal architecture reconstructed using SciClone, showing evolutionary relationships between subpopulations (SPs) in G2 (blue) and G4 (red). Internal nodes represent hypothetical ancestral clones; external nodes reflect the cellular prevalence of each SP. **(C)**. Single-sample gene set enrichment analysis (ssGSEA) of curated immune and signaling pathways using RNA-seq expression data from G2 and G4. Normalized enrichment scores (NES) are shown; red and blue indicate positive and negative enrichment, respectively. **(D)**. Pathway enrichment of Egenes associated with significantly hypomethylated CpG sites in G4. Enrichment was assessed using the hypergeometric test, and p-values are indicated.

Clone evolution analysis suggested a common germline foundation, with phylogenetic divergence supported by MAF distributions and distinct subclonal mutations. Among these, *CIC* (T761LfsTer163), a frameshift mutation introducing a premature stop codon, and *BRCA2* (M3217V), a missense mutation replacing methionine with valine, showed increased representation in grade 4, indicating clonal expansion (**Figure 3A**). The inferred phylogeny revealed common ancestral mutations at the trunk and lineage-specific alterations in each region (**Figure 3B**). Notably, 43 mutations were exclusive to grade 4, including *CIC*, *RPA4*, *BRCA2*, *NAF*, and *NOTCH1*, supporting the acquisition of additional oncogenic events during malignant progression.

Single-sample gene set enrichment analysis (ssGSEA) was performed using the GSVA package (v1.44.0) with curated immune-related gene sets from the MSigDB database (v7.5.1). Each gene set’s enrichment score reflects the normalized difference in empirical cumulative distribution functions (ECDFs) between genes in the gene set and the remaining genes in the sample. This analysis revealed upregulation of the MSP-RON signaling pathway and the Hinata NF-κB–associated immune inflammation signature in the grade 4 region. In contrast, WNT LRP6 and IL-17 signaling pathways were downregulated (**Figure 3C**). The IL-21 Up V1 gene set, associated with T follicular helper (Tfh) cell activation, also showed enrichment in grade 4 (**Figure 3D**). Additionally, gene ontology (GO) enrichment analysis was conducted using the clusterProfiler package (v4.6.0). Genes upregulated in grade 4 versus grade 2 (n = 3,362, fold change >1.5, p < 0.05) were tested for over-representation in GO biological process and molecular function categories. P-values were calculated using the hypergeometric test and adjusted using the Benjamini–Hochberg false discovery rate (FDR) method. Significantly enriched terms (adjusted p < 0.05) included acute inflammatory response, neuropeptide signaling, and regulation of coagulation (**Supplementary Figure S1**). All results are exploratory due to the single-patient design and require validation in larger cohorts.

**Figure 4:**
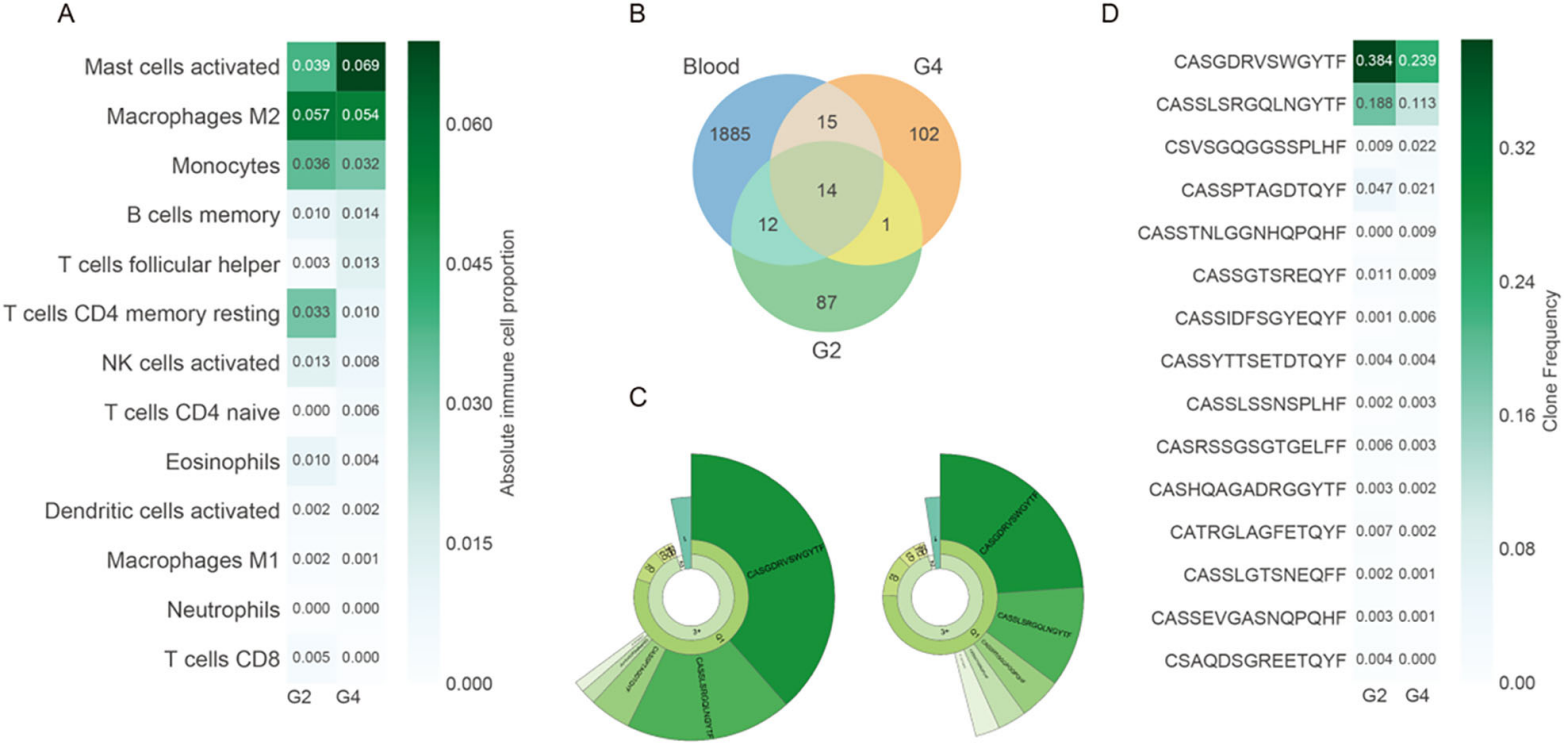
Immune microenvironment alterations during tumor progression. **(A)** Estimated absolute proportions of 13 immune cell types in G2 and G4 tumors using CIBERSORTx based on bulk RNA-seq data. Cell types are ordered by mean abundance. **(B)** Venn diagram showing the shared and unique T-cell receptor (TCR) clonotypes between G2, G4, and peripheral blood. **(C)** Donut chart representing clonal expansion in G2 and G4. The fan-shaped area indicates the clonal frequency. The radians of 1, 2, and 3+ represent the total frequency of TCR sequences with 1, 2, and 3 or more reads, respectively. The top 5 complementary determination region 3 (CDR3) amino acid sequences of TCR clones are displayed, with larger radians indicating higher clone frequencies. **(D)** Clone frequencies of the 15 shared clonotypes between G2 and G4. The greener colors represent higher clone frequencies. All immune cell and clonotype comparisons are descriptive and exploratory. Statistical testing was not performed due to the single-patient nature of this study.

Immune profiling based on deconvoluted RNA expression indicated an increase in activated mast cells (from 3.9% to 6.9% in grade 4), with concomitant decreases in resting CD4^+^ memory cells and CD8^+^ T-cell infiltration (**Figure 4A**). T-cell receptor (TCR) sequencing revealed 1,926 unique clonotypes in peripheral blood, with only 42 (2.13%) detected in either tumor region (**Figure 4B**). The Shannon diversity index was higher in grade 4 (3.76) than in grade 2 (3.01), while clonality declined (0.50 in grade 2 vs. 0.36 in grade 4), suggesting increased TCR diversity potentially reflecting neoantigen emergence. Fifteen TCR clonotypes were shared between grade 2 and grade 4, all of which showed reduced frequencies in grade 4, consistent with diversification (**Figure 4C**).

Two clonotypes with the highest clone frequency, CASGDRVSWGYTF and CASSLSRGQLNGYTF, were present in grade 2, grade 4, and peripheral blood (**Figures 4C, D**), indicating clone persistence and expansion. VDJdb alignment identified four TCR clonotypes with known viral specificities: three against cytomegalovirus (CMV) and one against Epstein–Barr virus (EBV), the latter comprising 1.44% of the grade 4 repertoire (**Supplementary Table S1**). All results are descriptive and exploratory, and no formal statistical significance testing was conducted due to the single-patient design. These findings provide preliminary insights into the genomic evolution, immune remodeling, and pathway alterations associated with untreated progression from grade 2 to grade 4 astrocytoma.

## Discussion

We report a case of a 67-year-old woman who carried an astrocytoma, IDH-mutant, CNS WHO grade 2, for over eight years without any surgical or pharmacologic intervention. This case is notable for the development of a spatially adjacent astrocytoma, IDH-mutant, CNS WHO grade 4, providing a rare opportunity to investigate the natural, untreated progression of IDH-mutant astrocytoma. Phylogenetic and molecular analyses confirmed that both tumor regions shared a common clonal origin, supporting the conclusion that the grade 4 astrocytoma evolved from the original grade 2 lesion.

The acquisition of additional mutations, including *CIC*, coincided with malignant progression and clonal expansion. Previous studies have implicated *CIC* alterations and activation of the RTK–RAS–PI3K pathway in recurrent high-grade gliomas (11, 12). One study has shown that 68% of recurrent glioma cases exhibited alterations in the RAS–ERK and PI3K–AKT pathways, often associated with treatment exposure (12). Our case supports the notion that RTK-RAS-PI3K pathway activation may also drive progression in untreated tumors. Notably, we observed enhanced inflammatory signaling and suppressed adaptive immunity accompanying progression. Compared to IDH wild-type lower-grade gliomas, IDH-mutant gliomas are associated with reduced expression of genes related to cytotoxic T cells and IFN-γ–inducible chemokines (13–15). In our case, hyperexpanded TCR clonotypes were detected. Although such expansions may reflect antigen-driven responses, three of the four dominant clonotypes matched viral epitopes (CMV, EBV), suggesting they may not be tumor-specific. Therefore, their relevance for immunotherapy remains uncertain in the absence of functional validation. Mohme et al. have found that dominant TCR clonotypes occupied substantial proportions of tumor-infiltrating lymphocytes in recurrent high-grade gliomas, with some clonotypes exceeding 15% frequency (16). In our case, the top two clonotypes reached 38% and 19% in grade 2 and 24% and 11% in grade 4, respectively. This may reflect prolonged tumor antigen exposure in a long-term tumor carrier. While the increased recruitment of mast cells and M2 macrophages during grade 4 progression is intriguing, this single-patient case is insufficient to establish causality or statistical significance. Observations regarding immune suppression, clonal evolution, and pathway involvement, such as MSP-RON and NF-κB signaling, should be considered hypothesis-generating. Larger cohorts with statistical modeling and mechanistic validation are needed.

Additionally, the absence of matched normal tissue limits our ability to distinguish somatic from germline variants. For example, the *BRCA2* (M3217V) may represent a germline variant misclassified as somatic. While we cannot exclude this, the relatively high mutant allele frequency and localization within functional domains suggest potential biological relevance. Previous studies have reported both somatic and germline *BRCA2* alterations in glioma, implicating this gene in DNA repair dysregulation (17). Nevertheless, in the absence of germline controls, mutational interpretations remain preliminary and require validation in future cohort studies. Furthermore, the absence of matched normal tissue may contribute to the elevated number of mutations detected in both tumor regions. Future studies incorporating normal-tissue controls and larger cohorts are essential to reliably distinguish somatic mutations and assess the significant immune cell dynamics in astrocytoma progression.

While our study used MRI for anatomical localization and clinical assessment, recent studies have applied radiomics-based analysis of MRI features to enhance glioma stratification. For example, a transfer learning–based model trained on MRI data from patients with astrocytoma, IDH–wildtype, CNS WHO grade 4, showed prognostic utility in IDH-mutant lower-grade gliomas (18). Additionally, XGBoost-based radiomics models have accurately predicted 1p/19q codeletion status in astrocytoma, IDH-mutant, and oligodendroglioma cases (19). These approaches highlight the complementary role of quantitative imaging in supporting non-invasive glioma classification alongside molecular profiling.

## Conclusion

This case illustrates the untreated clinical progression of an IDH-mutant grade 2 astrocytoma into a grade 4 astrocytoma in the absence of therapeutic intervention. Molecular profiling confirmed a shared clonal origin, with *CIC* and *BRCA*2 mutations potentially contributing to malignant progression. Mutations in *IDH, TP53*, and *ATRX* were observed in both regions, supporting their role in early gliomagenesis. The grade 4 lesion exhibited enhanced inflammatory signals, reduced T-cell infiltration, and hyperexpanded TCR clonotypes, which may have implications for immune surveillance and potential immunotherapy. Although limited by its single-patient design, this case provides valuable insight into untreated glioma evolution and highlights candidate mechanisms that warrant validation in larger cohorts.

## Data Availability

The original contributions presented in the study are included in the article/**Supplementary Material**. Further inquiries can be directed to the corresponding author.

## Supplementary material

Supplementary Figure 1 GO enrichment analysis of differentially expressed genes in grade 4 vs. grade 2 astrocytoma. Gene Ontology (GO) enrichment analysis was performed on differentially expressed genes (DEGs) between the grade 4 and grade 2 astrocytoma regions. Upregulated genes (n = 3,362; fold change > 1.5; p < 0.05) and downregulated genes were analyzed separately using the clusterProfiler R package. Enrichment was conducted for Biological Process (BP) and Molecular Function (MF) categories. P-values were calculated using a hypergeometric test and adjusted using the Benjamini–Hochberg false discovery rate (FDR) method. Dot size represents the number of DEGs per GO term, and color indicates the adjusted p-value.
