# Supplementary figures and images for: A case report on IDH-mutant astrocytoma, CNS WHO grade 4: multi-omic characterization of untreated clinical progression
Source: Front Oncol. 2025 Sep 26;15:1557245. doi: 10.3389/fonc.2025.1557245 (PMC12511056; doi:10.3389/fonc.2025.1557245)

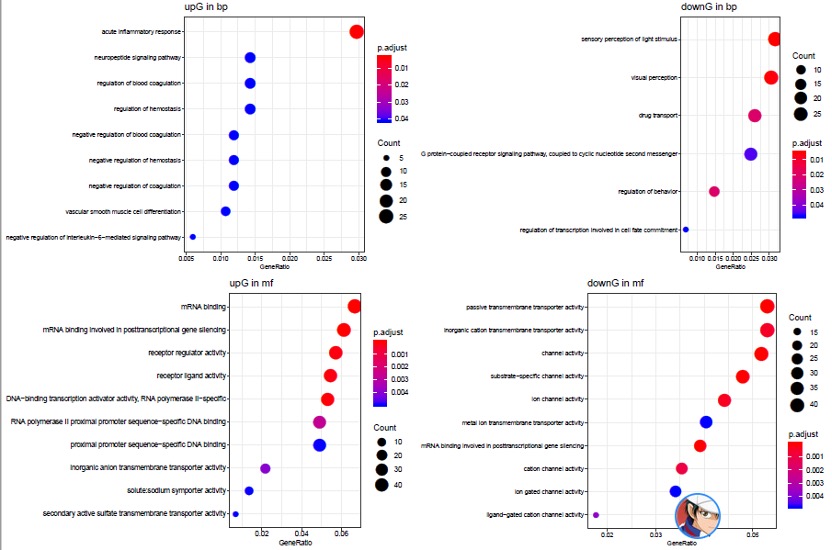

Supplement: Supplementary Figure 1 — GO enrichment analysis of differentially expressed genes in grade 4 vs. grade 2 astrocytoma. Gene Ontology (GO) enrichment analysis was performed on differentially expressed genes (DEGs) between the grade 4 and grade 2 astrocytoma regions. Upregulated genes (n = 3,362; fold change > 1.5; p < 0.05) and downregulated genes were analyzed separately using the clusterProfiler R package. Enrichment was conducted for Biological Process (BP) and Molecular Function (MF) categories. P-values were calculated using a hypergeometric test and adjusted using the Benjamini–Hochberg false discovery rate (FDR) method. Dot size represents the number of DEGs per GO term, and color indicates the adjusted p-value. [file Image1.jpeg]
