# Supplementary material for: A case report on IDH-mutant astrocytoma, CNS WHO grade 4: multi-omic characterization of untreated clinical progression
Source: Front Oncol. 2025 Sep 26;15:1557245. doi: 10.3389/fonc.2025.1557245 (PMC12511056; doi:10.3389/fonc.2025.1557245)
Supplement: Supplementary file 2 [file Table1.docx]

**Table S1.** Features of the four clonotypes that have matched records in VDJdb.

| **Sample ID** | **Clone Frequency** | **Clone Count** | **CDR3aa** | **V** | **J** | **MHC A** | **MHC B** | **MHC class** | **Epitope** | **Epitope species** |
| --- | --- | --- | --- | --- | --- | --- | --- | --- | --- | --- |
| G4 | 1.44% | 96 | CASSPGGAYNSPLHF | TRBV19 | TRBJ1-6 | HLA-A*02:01 | B2M | MHCI | GLCTLVAML | EBV |
| G4 | 0.08% | 5 | CASSLGTAYEQYF | TRBV11-2 | TRBJ2-7 | HLA-A*03:01 | B2M | MHCI | KLGGALQAK | CMV |
| G2 | 0.94% | 61 | CAWSVGGAGNTIYF | TRBV30 | TRBJ1-3 | HLA-A*02 | B2M | MHCI | NLVPMVATV | CMV |
| G2 | 0.11% | 7 | CASSERRGDEQFF | TRBV6-4 | TRBJ2-1 | HLA-A*03:01 | B2M | MHCI | KLGGALQAK | CMV |

Abbreviations: MHC, major histocompatibility complex; EBV, Epstein-Barr virus; CMV, cytomegalovirus
